# Reduced Ventricular Arrhythmogeneity and Increased Electrical Complexity in Normal Exercised Rats

**DOI:** 10.1371/journal.pone.0066658

**Published:** 2013-06-18

**Authors:** Horesh Dor-Haim, Omer Berenfeld, Michal Horowitz, Chaim Lotan, Moshe Swissa

**Affiliations:** 1 Heart Institute, Hadassah-Hebrew University Medical Center, Jerusalem, Israel; 2 Center for Arrhythmia Research, University of Michigan, Ann Arbor, Michigan, United States of America; 3 Department of Physiology, Hadassah-Hebrew University Medical Center, Jerusalem, Israel; 4 Cardiac Research Center, Kaplan Medical Center, Rehovot, Israel; Brigham & Women's Hospital - Harvard Medical School, United States of America

## Abstract

**Background:**

The mechanisms whereby aerobic training reduces the occurrence of sudden cardiac death in humans are not clear. We test the hypothesis that exercise-induced increased resistance to ventricular tachycardia and fibrillation (VT/VF) involve an intrinsic remodeling in healthy hearts.

**Methods and Results:**

Thirty rats were divided into a sedentary (CTRL, n = 16) and two exercise groups: short- (4 weeks, ST, n = 7) and long-term (8 weeks, LT, n = 7) trained groups. Following the exercise program hearts were isolated and studied in a Langendorff perfusion system. An S_1_–S_2_ pacing protocol was applied at the right ventricle to determine inducibility of VT/VF. Fast Fourier transforms were applied on ECG time-series. In-vivo measurements showed training-induced increase in aerobic capacity, heart-to-body weight ratio and a 50% low-to-high frequency ratio reduction in the heart rate variability (p<0.05). In isolated hearts the probability for VF decreased from 26.1±14.4 in CTRL to 13.9±14.1 and 6.7±8.5% in the ST and LT, respectively (p<0.05). Duration of VF also decreased from 19.0±5.7 in CTRL to 8.8±7.1 and 6.0±5.8 sec in ST and LT respectively (p<0.05). Moreover, the pacing current required for VF induction increased following exercise (2.9±1.7 vs. 5.4±2.1 and 8.5±0.9 mA, respectively; p<0.05). Frequency analysis of ECG revealed an exercise-induced VF transition from a narrow single peak spectrum at 17 Hz in CTRL to a broader range of peaks ranging between 8.8 and 22.5 Hz in the LT group (p<0.05).

**Conclusion:**

Exercise in rats leads to reduced VF propensity associated with an intrinsic cardiac remodeling related to a broader spectral range and faster frequency components in the ECG.

## Introduction

Many epidemiological studies support the concept that regular exercise correlates with reduced cardiovascular and overall mortality in apparently healthy individuals [1]–[4] and in patients with cardiovascular disease [5]. Recently, Kokkinos et al [6] demonstrated an inverse and graded relationship between exercise capacity and all-cause mortality in humans. Mozaffarian et al [7] further shows that light to moderate physical activities are associated with significantly lower atrial fibrillation (AF) incidence in older adults. Epidemiologic studies also document that regular physical activity reduces the incidence of ventricular arrhythmias [8]–[10] and in particular the incidence of SCD [11]. The protective effect of exercise was also demonstrated in animal models. Billman et al [12] show that daily exercise prevented VF induced by acute myocardial ischemia in a subpopulation of dogs that were identified as susceptible to SCD. They also demonstrate that exercise altered the autonomic tone toward the parasympathetic arm. Posel et al [13] revealed that exercise training increases the ventricular threshold of the previously infracted isolated rat heart before and after the onset of re-infarction. This effect was attributed again to altered autonomic tone toward the parasympathetic arm. Furthermore, it was shown that exercise training that was accompanied by a shift toward increased vagal activity reduces mortality in post-MI patients [14]. In addition, it was shown that stimulation of the left stellate ganglion in dogs is correlated with both ventricular arrhythmia [15] and atrial arrhythmia [16]. Furthermore, ablation of cardiac sympathetic neurons reduces the susceptibility to ischemia-induced sustained ventricular tachycardia in rats [17].

Despite the clear association of regular exercise programs with a reduction in the occurrence of atrial and ventricular arrhythmias, the manner in which exercise promotes these beneficial effects remains inadequately defined. Thus, the aim of this study is to characterize the influence of an aerobic exercise program on the cardiac structure and the function in healthy rats, in order to test the hypothesis that the properties of ventricular arrhythmias are altered intrinsically to the heart with the increased aerobic capacity. To achieve our goal we subjected rats to varying levels of exercise training programs and measured in-vivo heart rate variability as well as their cardiac mechanical function, ventricular arrhythmia susceptibility and spectral content of the induced VF in their isolated hearts. Our results demonstrate that increased aerobic capacity and reduced susceptibility to VT/VF are linked to an increase in temporal variability of the heart rate. In the isolated hearts we further demonstrate an intrinsic exercise-induced increase in the complexity of the temporal and spectral excitation patterns during the arrhythmias, independent from the nervous system function.

## Methods

### Animals and Exercise Protocol

Thirty male rats (Sprague-Dawley, weight 250–300 g) were studied. The study protocol was approved by the Ethics Committee of the Medical school at the Hebrew University, Jerusalem, in compliance with the American Heart Association guidelines. The rats were randomly divided into three groups: A control group (CTRL, n = 16) which remained sedentary for 8 weeks; a short term (ST, n = 7) trained group which remained sedentary for 4 weeks and then exercised for 4 weeks; a long term (LT, n = 7) trained group which exercised for 8 weeks. The timeline and details of the exercise program are summarized in table 1.

**Table 1:** Exercise protocol for the short term (ST) and long term (LT) trained groups.

	Week	1	2	3	4	5	6	7	8	9
ST	Exercise duration (min)	5	0	0	0	5	25	30	35	40
	Speed (m/min)	12	0	0	0	12	14	15	16	18
	Slope (degree)	0	0	0	0	0	0	4	6	8
LT	Exercise duration (min)	5	25	30	35	40	45	50	55	60
	Speed (m/min)	12	14	15	15	16	16	17	17	18
	Slope (degree)	0	0	0	2	2	4	4	6	8

(Note the increased duration and intensity of the exercise in the trained groups).

### In-vivo Monitoring

An aerobic test was done twice at the beginning (week 1) and at the end (week 8) of the study (see protocol, table 2). A twenty-minute ECG recording was collected (at weeks 1 and 8) for heart rate (HR) and heart rate variability (HRV) measurements. Three electrodes were fixed to the rats' skin. The fixation of the electrodes to the rats was done one week before the recording of the ECG. Rats were anesthetized with ketamine 85 mg/kg and xylazine 15 mg/kg. Metal clips were attached to animals skin in 3 spots, at the lower back besides the base of the tail and at the left and the right front legs just under the scapula. The ECG recording done in the rats cage at regular environment, while rats were not restricted, free to walk and to interact with other rats in the cage. The measurements were always taken at the same time of the day (17:00–19:00), with constant lightening conditions. One ECG signal was recorded, while the first 5 min were defined as adaptation time and were excluded. The ECG recording was obtained with power lab 5 AD Instrument and filtered at band pass 60–100 Hertz. The HR and HRV measurements were obtained using HRV Analysis Software v1.1 SP1[18] (The Biomedical Signal Analysis Group, University of Kuopio, Finland).

**Table 2:** Aerobic test protocol at weeks 1and 8 of the study.

Time (min0	1	2	3	4	5	6	7	8	9	10	11	12	13	14	15	16	17	18	19	20
Speed (m/min)	12	13	14	15	16	17	18	19	20	21	22	23	24	25	26	27	28	29	30	31

### The Langendorff Perfusion System

On day 75, the hearts were excised for mechanical and electrophysiological measurements. The animals were anesthetized, the chest was opened and the heart was quickly removed and placed in a solution composed of cold saline and heparin. The heart was connected to a Langandorff system and perfused with modified Krebs solution (The modified Krebs-Hensleit solution contents was as follows: 118 mM NaCl, 4.9 mM KCl, 2.3 mM MgSO_4_.7H_2_O, 25 mM NaHCO_3_, 1.2 mM KH_2_PO_4_, 2.5 mM Ca^+2^ and 11.1 mM glucose), at temperature of 37°C within 120 seconds.

### Isolated Heart Monitoring

Two platinum electrodes were placed on the left ventricle apex and the right atrial appendage to obtain an ECG at a sampling rate of 2000 Hz. Left ventricular pressure (LVp) was recorded using a pressure transducer inserted into the left ventricle (highly accurate and robust piezo-resistive transducer, MLT844 Physiological Pressure Transducer, ADInstruments). ECG and pressure were acquired into a PowerLab data acquisition system (AD Instruments Inc, Mountain View, CA, USA). The cardiac output was indirectly determined based on the time integral of LVp. Systolic function was determined as (dLVp/dt)_max_ and diastolic function as (dLVp/dt)_min_.

### Electrophysiological Protocol

Two additional electrodes were placed on the free wall of the right ventricle for pacing and stimulation using a programmable stimulator (Master 8, Israel). A unified anatomical placing of the electrodes was kept as follows: one RV pole was placed 4–5 mm under the right atrial appendage and the second RV pole was place horizontally 4 mm apart. The pacing threshold was determined at 5 Hz. Two stimulation protocols were used to determine the effective refractory period (ERP) and VT/VF induction threshold. VT was defined as a rapid rate with changed QRS morphology from the basic rate but with a new mono-morphic pattern. VF was defined as a rapid rate with randomly changed complex morphology. ERP was measured using the standard S1–S2 protocol at twice the pacing threshold intensity. A set of 16 sequences each containing 8 beats at a CL of 260 ms (S1), and a 9^th^ beat (S2) with a coupling interval of 100, 90, 80, 70, 60, 55, 50, 45, 40, 35, 30, 25, 20, 15, 10, and 5 ms was used for induction of VT/VF. The initial pacing intensity was set at twice the pacing threshold and a pulse width of 1 ms for both S1 and S2. The S1–S2 sequences were applied until induction of VT/VF or loss of capture was detected. If the index set did not induce VT/VF, a new set of 16 sequences was repeatedly applied (after a rest period of 60 seconds) with increasing pacing intensity of S2 only (0.2 mA up to 10 mA) The VT/VF thresholds were defined as the minimal pacing intensity that induces the arrhythmias. The VT/VF coupling intervals were defined as the longest S_2_ inducing the arrhythmias. The distribution of the coupling intervals was determined as the standard deviation of those S_2_. Once an induced arrhythmia was sustained for more than 60 seconds, intra-coronary lidocaine hydrochloride (0.25 mg) was used for chemical defibrillation. A washout period of 15 minutes was applied before introducing the next stimulation protocol. Probability for arrhythmia was determined as the number of induced VT/VF per 1000 attempts.

### Spectral Analysis of VF

All ECG recordings of sustained VF (>2.5 sec, the first 0.5 sec ignored) were spectrally analyzed to determine their frequency content using fast Fourier transform (FFT, Power Lab Chart 5 software). Accordingly, 10–20 second episodes of contiguous VF were segmented into sequential sections of 1-second long each. Subsequently the 1 second-long sections were zero padded and their power spectra calculated separately using a 0.24 Hz resolution FFT. Finally, the individual power spectra were averaged through the Welch method [19] for each animal and for all the animals in their respective groups in order to obtain an overall frequency content characterization. Dominant frequency (DF) was defined as the frequency with maximal power in the spectrum.

### Statistics

Values are expressed as mean±standard deviation. An unpaired student's t-test was used to assess the differences between groups. Categorical data was compared using a chi-square test. Differences were considered significant at p<0.05.

## Results

### Aerobic Exercise Capacity and Animal Survival

The aerobic exercise capacity increased significantly in both ST and LT groups as compared with the CTRL group (p<0.01, Table 3). The survival rate for the full stimulation protocol (see Electrophysiological Protocol part in the method section) was 100%, 71% and 34% for LT, ST and CTRL group respectively (p<0.0001). The positive effect of the exercise appeared within the first 4 weeks of training (ST group) and continued to increase in the LT group.

**Table 3:** The effect of exercise on the in vivo (aerobic exercise capacity) and in vitro (cardiac output, systolic and diastolic function) measurements.

		CTRL	ST	LT	p Value
		n = 16	n = 7	n = 7	
Exercise Aerobic Capacity (min)	Day 7	10.64±0.22	10.72±0.38	9.33±0.4	
	Day 73	9.58±0.1	11.88±0.28	12.17±0.65	
	Percentage change	−9%	+11%	+30%	<0.01
Mechanical functions of isolated hearts	Cardiac output (mmHg/min)	26555±854	31086±1657 (+19%)	31168±854 (+19%)	<0.05
	Systolic function (mmHg/sec)	4064±230	4123±421	5462±532 (+34%)	<0.05
	Diastolic function (mmHg/sec)	−2994±78	−3392±683 (+13%)	−3437±283 (+15%)	NS

CTRL, control group; ST, short term trained group; LT, long term trained group.

### Exercise and the Structure of the Heart

The body weight of the rats at the end of the study was 359±15, 324±6 and 334±16 grams for CTRL, ST and LT groups respectively (p = NS). The heart weight was 1.47±0.08, 1.51±0.06 and 1.54±0.04 grams for CTRL, ST and LT groups respectively (ST vs. CTRL: p = 0.05; LT vs. CTRL p = 0.001). The heart to body weight ratio (×1000) was higher in the ST and LT groups compared with CTRL (4.6±0.6 4.7±0.3 and 4.1±0.1, respectively; p<0.05). The heart diameter remained the same for the CTRL, ST and LT groups (11.7±0.4, 12±0.4, and 11.7±0.2 respectively). However, the thickness of the LV wall was greater in the LT group (4.4±0.1 mm; p = 0.001) as compared with CTRL (3.8±0.2 mm).

### In-Vitro Mechanical Functions

A significant increase in cardiac output (20%; p<0.05) was observed in both ST and LT groups compared with CTRL group (Table 3). The systolic function increased by 34% in the LT group compared with CTRL group (p<0.05). The diastolic function presented a positive trend in both the ST and LT (Table 3).

### Heart Rate and Heart Rate Variability

Twenty minute in-vivo recordings of the ECG at weeks 1 and 8 were used for HR and HRV measurement. The mean HR at week 1 was comparable in all groups. However, at week 8 the mean HR was significantly lower in the LT group compared with control and ST groups (Table 4). In addition, the HR in the LT group was significantly lower on week 8 compared with week 1. However, in the isolated hearts the mean HR was lower in the CTRL hearts compared to ST and LT hearts (Table 4). See the discussion for an interpretation of these results.

**Table 4:** Heart rate (bpm) at the the different phase of the study.

Heart rate in:	CTRL	ST	LT	p Value
	n = 16	n = 7	n = 7	
Week 1	422±11	415±19	409±27^#^	NS
Week 8	411±30*	416±21*	381±34*^,#^	*, # <0.05
Isolated Heart	240±5**	253±3	262±8**	** <0.05

CTRL, control group; ST, short term trained group; LT, long term trained group.

**Figure 1:**
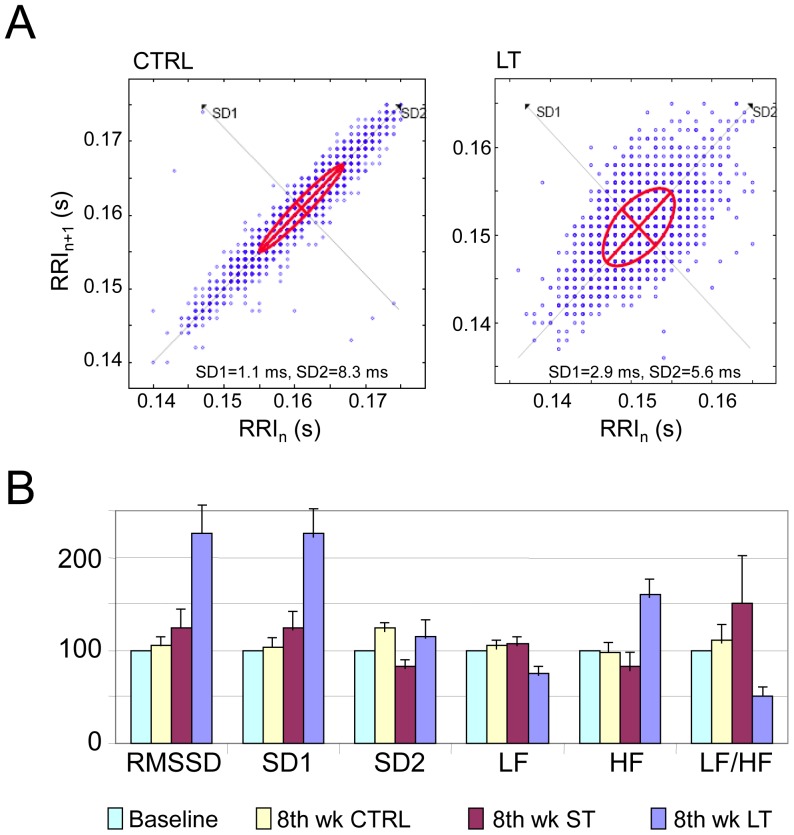
Heart rate variability measures. A. Poincare plots for sample CTRL and LT animals (comparison of ST group to CTRL was not significant, not shown). B. HRV parameters for the 3 groups relative to their normalized value at week 1 in %.

HRV data are presented in Figure 1 and Table 5. At baseline, the variance of the RR interval series as measured by the RMSSD in LT (1.9±0.5 ms) was comparable with that of the CTRL animals (2.1±0.5 ms; p = NS). In contrast, at week 8 the RMSSD of the LT group was 4.5±1.6 ms as compared with CTRL at 2.2±0.9 ms (p<0.05). This increase in variability was further analyzed by the dispersion of the RR values on Poincare return maps as shown in Figure 1A. The red ellipses superimposed on maps from two representative animals denote the standard deviation values across (SD1) and along (SD2) the unitary line representing short and long term variability in the RR values. The maps in Figure 1A for the 2 animals show clearly that while the SD1 increases in the LT animal compared with the CTRL animal, the SD2 does not. Table 5 shows that on average at week 8 the short term variability in the RR intervals is larger in the LT animals (SD1 = 3.2±1.1 ms) compared with the CTRL animals (SD1 = 1.63±0.4 ms, p<0.05). On the other hand, the long term variability as measured by SD2 does not vary between the groups or between weeks 1 and 8.

**Table 5:** Heart Rate Variability (HRV) measurements.

		RMSSD (ms)	SD1 (ms)	SD2 (ms)	LF (ms^2^/Hz)	HF (ms^2^/Hz)	LF/HF
CTRL	Baseline	2.1±0.5	1.6±0.4	6.7±1.6	68.5±7.7	30.2±7.3	2.45±1.1
	Week 8	2.2±0.9	1.63±0.4	8.5±1.3	69.8±9.6	29.5±9.2	2.7±1.3
ST	Baseline	1.8±0.5	1.38±0.4	7.4±1.9	71.5±10.8	27.4±11	3.13±1.7
	Week 8	2.3±1	1.7±0.7	6.1±1.3	76.6±11.7	22.8±11.8	4.72±3.9
LT	Baseline	1.9±0.5	1.4±0.4	7.5±2.7	66.9±7.8	31.9±8	2.32±0.9
	Week 8	4.5±1.6*	3.2±1.1*	8.6±5.4	47.4±19.2*	52.1±19.1*	1.13±0.8*

RMSSD, root mean square of successive differences; SD1, short-term HRV; SD2, long-term HRV; LF, low-frequency power; HF, high-frequency power; CTRL, control group; ST, short-term trained group; LT, long-term trained group.

* p<0.05.

**Figure 2:**
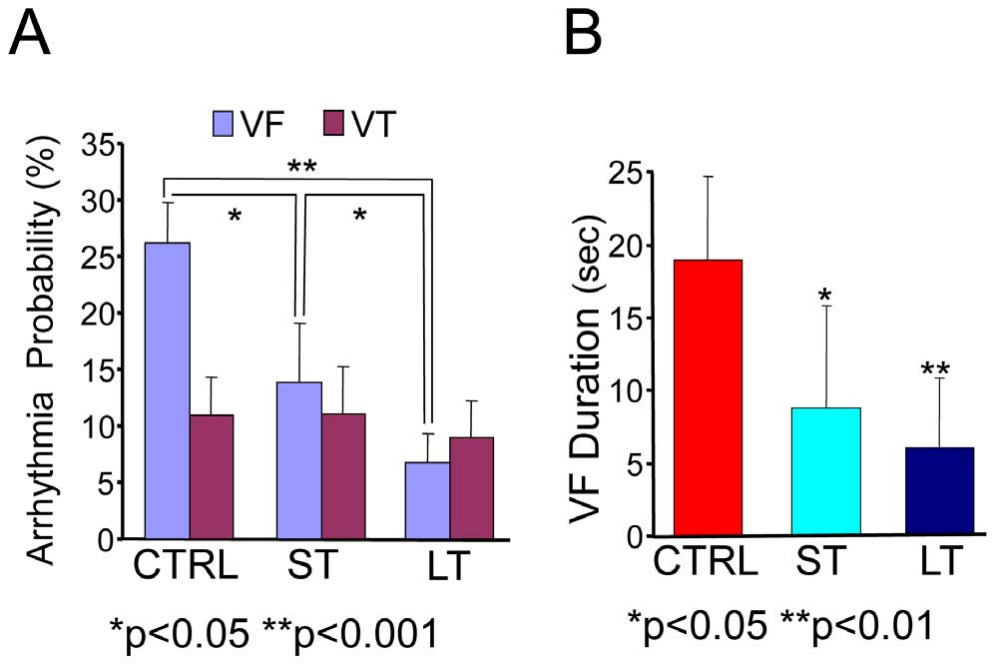
Probability and duration of VT/VF episodes. A. Probability for VT and VF induction as percentage number of events per 1000 stimuli. B. Duration of contiguous episodes of VF in the 3 groups (p<0.05 for ST to CTRL and p<0.01 for LT to CTRL).

The spectral analysis of the HRV shows a different time course for the RR intervals between the groups (Table 5 and Figure 2). Whereas the CTRL and ST groups show no significant alterations in the levels of the LF and HF power from week 1 to week 8, for the LT group the LF power decreased (from 66.9±7.8 to 47.4±19.2 ms^2^/Hz, p<0.05) and the HF components increased (from 31.9±8 to 52.1±19.1 ms^2^/Hz, p<0.05). This resulted in an approximate 50% LF/HF ratio reduction (p<0.05), which is compatible with an increased ratio of parasympathetic over sympathetic tone for the LT group, as seen also by the reduced HR in that group (see discussion for details).

### Inducibility of VT/VF

Arrhythmias were induced by programmed pacing in isolated hearts. The threshold current intensity for the pacing was comparable in all three groups (0.24±0.05, 0.2±0.04 and 0.24±0.06 mA for CTRL, ST and LT groups respectively; p = NS). ERP was also comparable between the three groups (49.5±11, 55.7±9.3 and 50±7.6 ms for CTRL, ST and LT, respectively; p = NS). VT/VF were induced by the S1–S2 stimulation protocol described above in 16/16 of the CTRL rats as compared with 5/7 and 4/7 in the ST and LT groups, respectively (p = 0.025 by contingency Chi-square test). To further analyze VT and VF reproducibility, the probability of the arrhythmias was calculated as the number of events of VF or VT for 1000 stimuli attempted. While the probability of induction of VT was comparable in the three groups (10.9±13.5, 11.1±10.6 and 8.9±12.3% for CTRL, ST and LT, respectively, p = NS), the probability for induction of VF was 2–4-fold higher (Figure 2A) in the CTRL group as compared with the ST and LT groups (26.1±14.4, 13.9±14.1 and 6.7±8.5%, respectively; p<0.05). The average duration of VF was significantly shorter (Figure 2B) in the trained groups when compared with the control group, (19.0±5.7, 8.8±7.1 and 6.0±5.8 sec for CTRL, ST and LT, respectively, p<0.05).

**Figure 3:**
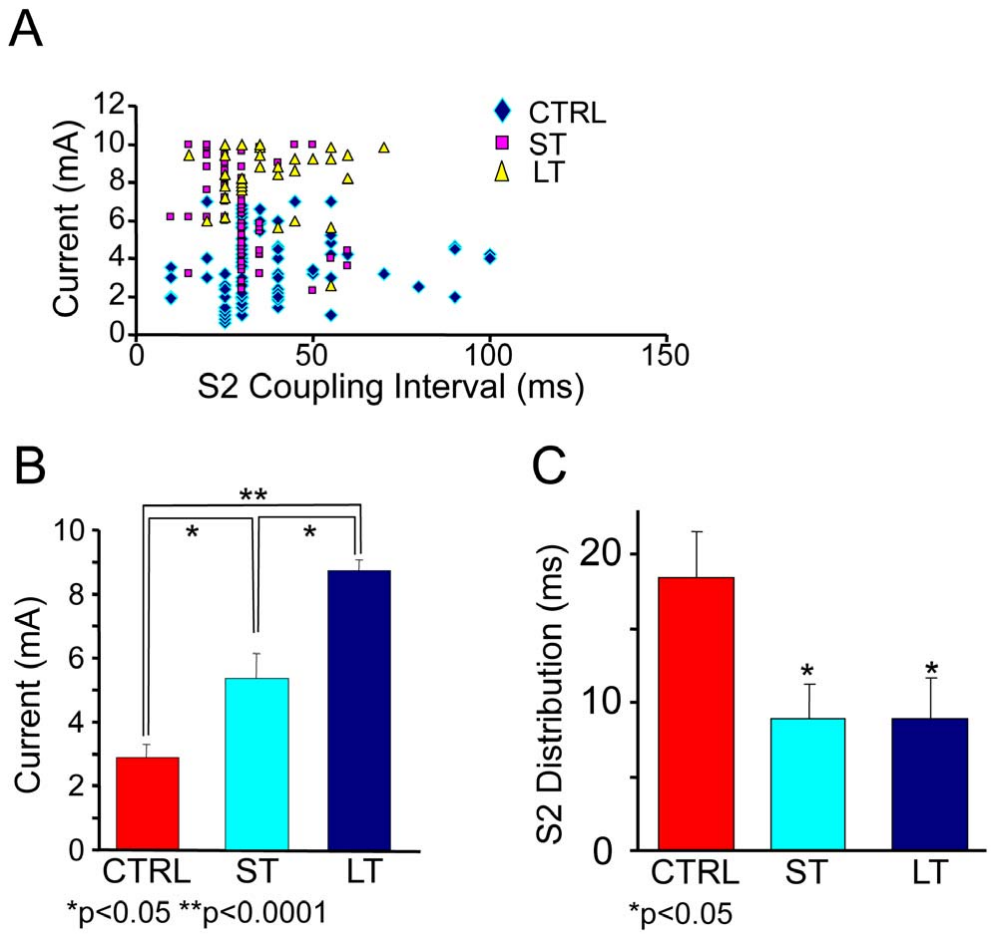
Induction of VF and exercise intensity. A. Current intensity and S_2_ interval for VF induction in individual experiments for the 3 groups. B. Threshold current for VF induction in the studied groups (p<0.05 for ST compared to LT and CTRL, p<0.0001 for LT compared to CTRL). C. The relationship between the exercise intensity (the different groups) and the distribution of stimulus coupling interval S_2_ that induces VF in the studied group (ST and LT compared to CTRL).

The threshold for arrhythmia induction followed a similar pattern. While no effect was observed on the threshold for induction of VT following exercise (not shown), a clear effect of exercise on VF induction was observed. In Figure 3A we show data on the current and the S2 coupling interval required for VF induction. The data show that the combination of a higher pacing intensity over less distributed coupling intervals was used for the induction of VF in the LT and ST groups as compared with the control group (most VF events were not preceded by VT). Quantification of the data in Figure 3A can be seen in Figure 3B as a significant increase in the pacing current with the duration of the exercise (2.9±1.7, 5.4±2.1 and 8.5±0.9 mA for CTRL, ST and LT groups, respectively; p<0.05 for CTRL vs. ST, or ST vs. LT; p<0.001 for CTRL vs. LT). In addition, the coupling interval that induces VF was significantly shorter in the ST and LT groups compared with CTRL group (Figure 3C). The distribution index (calculated as the average of the SD of the coupling interval distribution for each group) for the VF induction coupling interval was significantly higher in the control group as compared with the ST and LT groups (18.5±3.1, 8.8±2.3 and 8.9±2.8 ms for CTRL, ST and LT groups, respectively, p = 0.05). Overall it can be appreciated that for induction of VF in the exercised groups a more aggressive protocol was used which included the combination of a higher pacing current with a short coupling interval for the extra stimulus.

### Effect of Exercise on the Spectral Properties of VF

**Figure 4:**
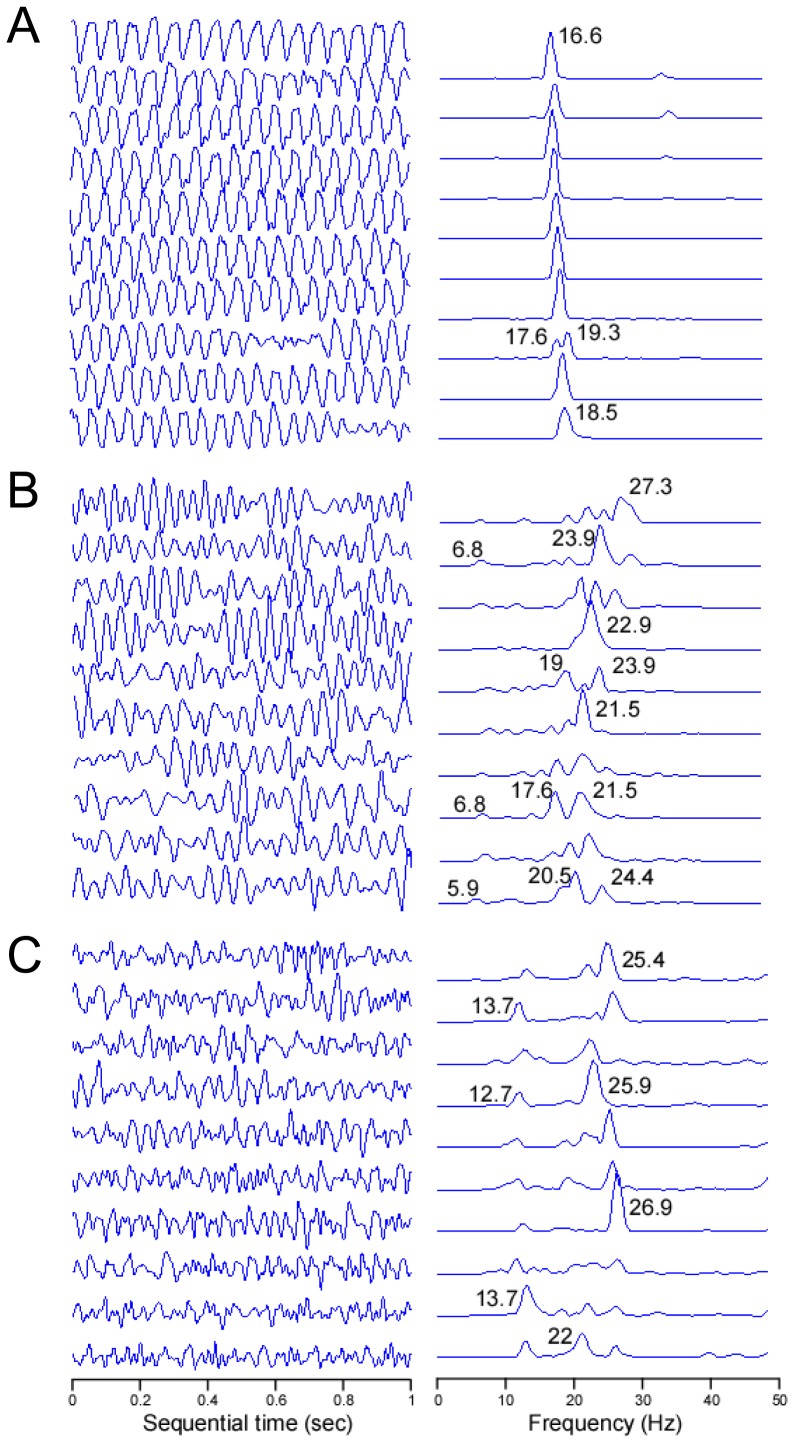
Time course of spectral properties of VF in a control and exercised sample rats. Ten consecutive 1-second long pseudo-ECG episodes of VF (from early at top to late at bottom) with their corresponding power spectra for sample animals from the CTRL (A), ST (B) and LT (C) groups, respectively. Numbers on power spectra traces indicate values of significant peaks in Hz.

**Figure 5:**
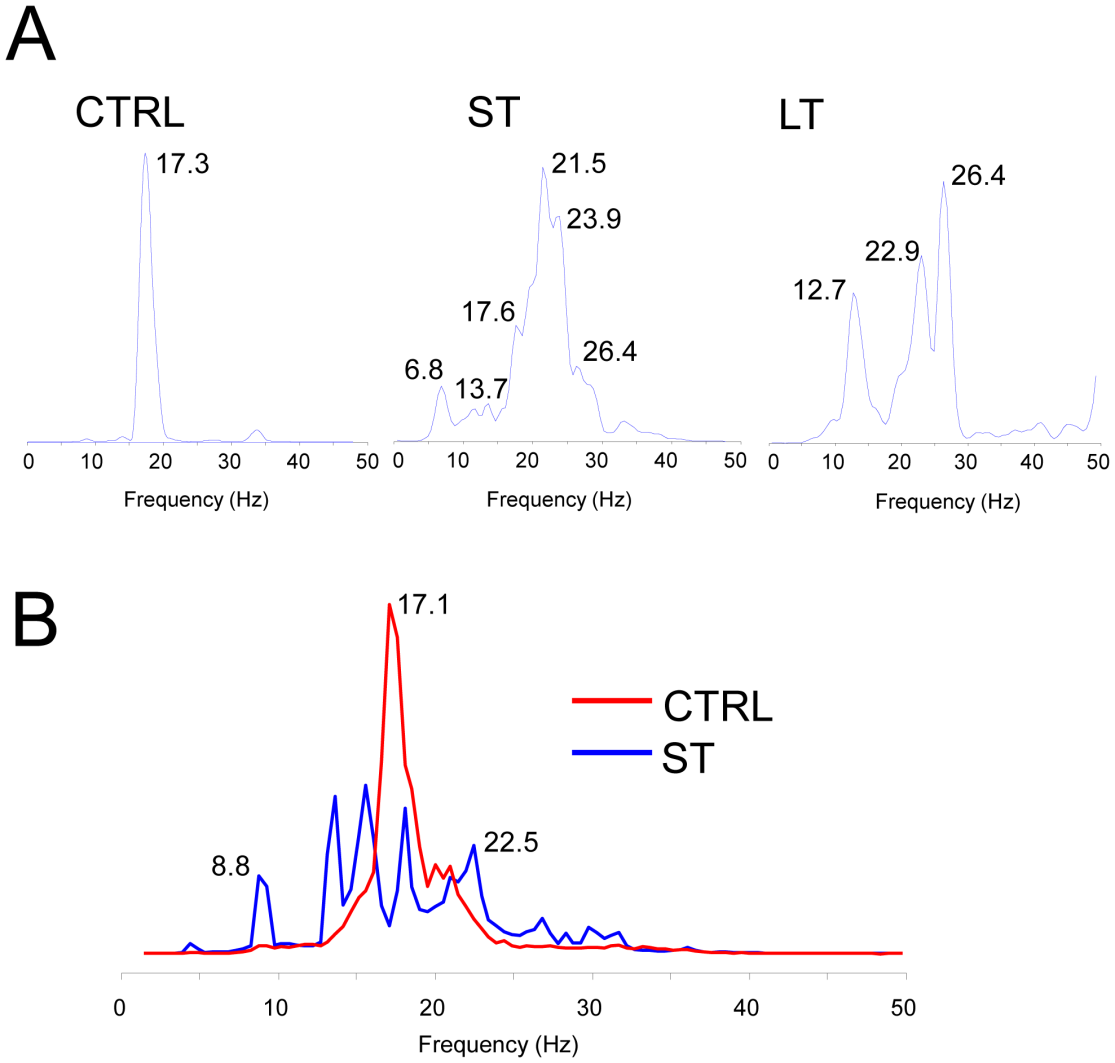
Welch power spectra. A. Welch averaged 10 consecutive power spectra of single animals from the CTRL (left), ST (middle) and LT (right) groups. B. Averaged power spectra of 1-second long pseudo-ECG segments for 93 VF events lasting more than 2 seconds in the CTRL (red) group and for 29 VF events lasting more than 2 seconds in the ST (blue) group (This analysis was not performed for the LT group because only 5 VF events in 4/7 animals were detected in that group).

Characterization of the VF in the frequency domain revealed for the first time a distinct profile for VF in control vs. exercised rats. Figure 4 shows the time course of 1-sec consecutive segments of pseudo-ECG measurements across the isolated hearts alongside their corresponding power spectra. The sequential control power spectra is seen to be mostly single peaked with a slight temporal variation in peak frequencies between 16.6 and 19.3 Hz (Figure 4A). In comparison, the power spectra for the 2 sample animals in the ST and LT groups are visibly more complex, both for each of the 1-sec segments and also in their time course; instead of a single and narrow spectral peak as observed in the control animal, the exercised animals had at any given moment multiple spectral peaks spanning a wider band between a lower frequency of 8.5 and a higher frequency of 26 Hz as compared with the control sample. Figure 5A shows the Welch-averaged power spectra for the 3 animals analyzed in Figure 4. It confirms that over the period of 10 seconds the control animal had a narrow spectrum with a DF of 17.3 Hz, and the ST and LT animals had more spectral components spanning a broader range with multiple peaks primarily below the DF peaks of 21.5 and 26.4 Hz, respectively, which are higher than the DF of the control. Figure 5B shows the distinct (p<0.01) averaged power spectra of the pseudo-ECGs for all VF events lasting at least 2 seconds that were recorded in all animals in the CTRL (93 VF events in 16/16 animals) and the ST groups (29 VF events in 5/7 animals). This analysis was not performed for the LT group because only 5 VF events in 4/7 animals were detected in that group. The averaging of the power spectra (see Methods) reveals a relatively narrow-peaked spectrum with a DF of 17 Hz in the control group (red). In contrast, the averaging of the power spectra for all the animals in the moderately exercised group (ST group) revealed a broader range of peaks ranging between 8.8 and 22.5 Hz.

## Discussion

The major finding of this study is that a continuous exercise program in normal rats is characterized by alterations in the properties of VF that are associated with alterations that are exclusively intrinsic to the heart. An increased resistance to VF in the isolated hearts of long-term trained vs. control rats was concluded based on: i) reduced probability for VF, ii) increased threshold current for VF induction, and iii) reduced duration of the induced VF. This resistance for arrhythmia induction was demonstrated in the past, especially in ischemic models [12]–[14], [17], [20], [21]. Here, however, we further provide insight into possible mechanisms underlying the shorter duration of VF in the exercised vs. control normal rats. We characterized HRV prior and during the arrhythmia and found that reduced VF duration correlated with increased short-term variations in RR intervals and a reduction in its LF/HF ratio. To our knowledge this is the first study using spectral analysis of ECG to demonstrate that the shorter episodes of VF in hearts from long-term trained animals are characterized by an intrinsic remodeling with broader spectral content and faster components as compared with control hearts.

### Exercise and Antiarrhythmic Mechanisms

The manner by which the exercise program contributes to mechanical and electrophysiological remodeling associated with cardio-protection against ventricular arrhythmia is unclear as of yet. Several mechanisms have been proposed for this benefit: i) Enhanced cardiac β_2_-adrenoceptor (β_2_-AR) responsiveness can increase susceptibility to VF. It was found that exercise training with autonomic neural remodeling reduced the β_2_-AR responsiveness and decreased the risk for VF [22]. ii) Adenosine receptor blockade with the non-selective adenosine receptor antagonist theophylline or aminophylline attenuated the cardio-protective effects of dynamic exercise, suggesting that strategies that lead to adenosine production provide protection against lethal arrhythmias [23]. iii) Daily exercise increased the ventricular arrhythmia threshold by altering calcium regulatory proteins such as a decrease in the protein expression of the Na^+^/Ca^2+^ exchanger and as normalization of the protein expression of phospholamban in the hypertensive rats [24]. and iv) Reduction in repolarizing K^+^ current density is evident in patients with pathologic cardiac hypertrophy [25], [26] and may underlie pro-arrhythmic changes (action potential prolongation and increased dispersion of repolarization) [27], [28]. However, in exercise-induced physiologic hypertrophy an upregulation of the repolarizing K^+^ current density parallels the increased myocyte size which may prevent arrhythmogenesis [29].

### Heart Rate and Heart Rate Variability Measurements

As shown in Table 4, the HR at week 8 was significantly lower in the LT group when compared both to LT group at week 1 and CTRL group at week 8. These results are compatible with the altered vagal activity toward the parasympathetic arm showed in the trained groups. However, in the isolated heart we measured an increased HR in the LT group as compared to the control group (Table 4). We thought that although the interruption of the autonomic activity appears in all isolated hearts, because the higher parasympathetic activity in the LT group in the in-vivo phase, once the interruption of autonomic activity took place the net balance toward the parasympathetic activity was lower in the LT group leads to higher HR.

In general, our HRV measures are compatible with those measured in small animals with very fast at-rest HR [30]. Spectral analysis of the sequence of the RR intervals quantifies the power level in two distinct bands of high (HF: 0.15–0.40 Hz) and low (LF: 0.04–0.15 Hz) frequencies associated with parasympathetic versus both sympathetic and parasympathetic tonus, respectively [31], [32]. An independent analysis of the nervous activity has been utilizing a Poincare return mapping of the RR intervals (Figure 1B). In those maps SD1 is a measure of the short term beat-to-beat changes influenced by the sympathetic to parasympathetic tonus ratio (comparable to RMSSD and HF). SD2 on the other hand is a measure of the overall dispersion of the RR intervals, and reflects long-term changes (comparable to LF). Our data demonstrate that the unequivocal increase in the in-vivo short-term HRV as measured independently by either RMSSD, SD1 or the HF components correlates with the reduced propensity and duration of VF in isolated hearts from rats undergoing an 8-week exercise program. The reduced long-term HRV, i.e. the LF, in the exercised animals further suggests an overall parasympathetic activity increase and sympathetic activity reduction in those animals. The link between the nervous system and the intrinsic cardiac remodeling following such an exercise program remains to be investigated.

### Exercise and Cardiac Hypertrophy

In our study the trained rats developed cardiac hypertrophy manifested by the higher heart weight and the heart-to-body weight ratio in both ST and LT groups compared to the control group. It is well established that while cardiac hypertrophy elicited by pathological (maladaptive) stimuli eventually leads to cardiac dysfunction [33], exercise–induced hypertrophy does not [34], [35]. Exercise training increases the cardiac mass, maximum oxygen consumption, coronary blood flow [36] and overall balanced growth of cardiomyocytes [37], [38]. It was also shown that the expression of marker genes differs between adaptive (exercise induced) and maladaptive (pathological induced) cardiac hypertrophy [39]–[41]. Recently, it was shown that exercise induces a significant down-regulation of the uncoupling protein 2 (UCP2) [42]. In contrast, in models of cardiomyopathy induced by chronic β-adrenergic signaling, the cardiac hypertrophy was associated with upregulation of UCP2 [43]. Furthermore, the upregulation of UCP2 was reversed by β-adrenergic blockade. These observations are compatible with our results – exercise induced cardiac hypertrophy was associated with resistance to cardiac arrhythmia induction, and this protective effect can be related partially to the change in the autonomic balance toward the parasympathetic arm as suggested by the HRV data measurements.

### Spectral Characteristics of VF

Spectral analysis of electrical activity and its activation rate as measured by the DF have been used to gain insight into mechanism underlying fibrillation [44]. In brief, regional heterogeneity in the DF of local electrical activity has been attributed to the presence of a driving reentrant source localized to the region with the highest DF and a particular ionic makeup [45]. The hierarchy of decreasing DFs at the periphery of the highest DF is said to result from frequency-dependant conduction impairments whereby the acceleration of a driver (i.e., higher DF) would increase the complexity of the global patterns of activity with an increased number of frequency components in the global ECG [46]. Our spectral analysis of pseudo-ECGs in Figures 4 and 5 shows that VF in the control group was characterized by a narrow power spectrum with DF at about 17 Hz that was stable over the course of the recordings. In contrast, multiple peaks with higher DFs at >20 Hz were found in the power spectra of the ST and LT groups during VF. In Figure 4 we show that for the exercise animals the DFs in the temporal sequence of the power spectra is less stable compared with the DF peak of the control animal which, in addition to the acceleration of the DF, could also underlie the increased number of peaks seen in the averaged power spectra for those groups [19]. Overall, our results suggest that the sources of the VF in the trained animals accelerate to produce higher DF values which in turn give rise to additional frequency components corresponding to intermittent blockade of impulses as well as alterations in the direction and velocity of their propagation across the ventricles [46]. The temporal alteration in the spectral profile as seen in Figure 4 further leads us to speculate that intrinsic cardiac remodeling in the exercised animals prevents the stabilization of the fast sources, which may also explain the increased resistance to the VF induction as shown in Figures 2 and 3. The global nature of the electrical recordings in our study precludes a more detailed study of the origins of the reduced propensity to and duration of the fibrillation.

### Limitations

There are several limitations to our study. First, we show that exercise increases the threshold for VF induction in normal rats and more work should be done to study the effect of exercise in diseased rats. Secondly, our electrical recordings during sinus rhythm and arrhythmia are global and cannot give high-resolution information on spatial patterns of excitation. Nevertheless, our HRV and spectral analyses provide a novel insight into the frequency domain characteristics of the antiarrhythmic effect of exercise with inference to less stable reentrant drivers. Finally, despite the obvious intrinsic remodeling inferred by our global results in the isolated hearts experiments, no structural and cellular mechanisms underlying our observations have been explored in the present study and those will have to be considered in the future.

### Conclusions and Future Perspectives

Our study confirms that exercise in normal rats leads to reduced VF propensity and finds that this increased protection is associated with an intrinsic cardiac remodeling related to a broader spectral range and faster frequency components in the ECG. The structural, ionic and molecular mechanism underlying our observation should be the aim of further extensive investigation. That investigation should include (i) intra-cardiac in-vivo study of the cardiac electrical function during sinus rhythm (SR) and VT/VF to better outline the possible remodeling of the autonomic nervous system in the protection against VT/VF, (ii) a wide-field, high-resolution, optical mapping of activation patterns during those rhythms in the isolated hearts should reveal the extent to which the activation transforms from, or into, more stable reentrant and blockade patterns of activity, and finally, (iii) isolated hearts should be also subjected to electrophysiology studies in isolated cells for characterization of ionic, protein and other sub-cellular (e.g. the mitochondria) remodeling as well as for possible structural (e.g., fibrosis) remodeling. Based on previous studies on fibrillation dynamics [47]–[50], a possible first target for investigation of the mechanisms underlying the observed acceleration in the VF in the ST and LT rats would be the various K^+^ currents and channels, and among them the I_K1_ should be the first target. Fibrosis is a main structural factor that also needs to be investigated, in particular with regard to the complex dependency of excitation wave conduction on density, architecture and heterogeneous intercellular coupling [51], [52].
